# Prevalence and genetic profiles of isoniazid resistance in tuberculosis patients: A multicountry analysis of cross-sectional data

**DOI:** 10.1371/journal.pmed.1003008

**Published:** 2020-01-21

**Authors:** Anna S. Dean, Matteo Zignol, Andrea Maurizio Cabibbe, Dennis Falzon, Philippe Glaziou, Daniela Maria Cirillo, Claudio U. Köser, Lice Y. Gonzalez-Angulo, Olga Tosas-Auget, Nazir Ismail, Sabira Tahseen, Maria Cecilia G. Ama, Alena Skrahina, Natavan Alikhanova, S. M. Mostofa Kamal, Katherine Floyd

**Affiliations:** 1 Global TB Programme, World Health Organization, Geneva, Switzerland; 2 Emerging Bacterial Pathogens Unit, Division of Immunology, Transplantation and Infectious Diseases, IRCCS San Raffaele Scientific Institute, Milan, Italy; 3 Department of Genetics, University of Cambridge, Cambridge, United Kingdom; 4 Centre for Tuberculosis, National Institute for Communicable Diseases, Johannesburg, South Africa; 5 National TB Reference Laboratory, National Tuberculosis Control Programme, Islamabad, Pakistan; 6 National TB Reference Laboratory, Research Institute for Tropical Medicine, Muntinlupa City, Philippines; 7 Republican Scientific and Practical Centre for Pulmonology and Tuberculosis, Minsk, Belarus; 8 Main Medical Department, Ministry of Justice, Baku, Azerbaijan; 9 National TB Reference Laboratory, Dhaka, Bangladesh; McGill University, CANADA

## Abstract

**Background:**

The surveillance of drug resistance among tuberculosis (TB) patients is central to combatting the global TB epidemic and preventing the spread of antimicrobial resistance. Isoniazid and rifampicin are two of the most powerful first-line anti-TB medicines, and resistance to either of them increases the risk of treatment failure, relapse, or acquisition of resistance to other drugs. The global prevalence of rifampicin resistance is well documented, occurring in 3.4% (95% CI 2.5%–4.4%) of new TB patients and 18% (95% CI 7.6%–31%) of previously treated TB patients in 2018, whereas the prevalence of isoniazid resistance at global and regional levels is less understood. In 2018, the World Health Organization (WHO) recommended a modified 6-month treatment regimen for people with isoniazid-resistant, rifampicin-susceptible TB (Hr-TB), which includes rifampicin, pyrazinamide, ethambutol, and levofloxacin. We estimated the global prevalence of Hr-TB among TB patients and investigated associated phenotypic and genotypic drug resistance patterns.

**Methods and findings:**

Aggregated drug resistance data reported to WHO from either routine continuous surveillance or nationally representative periodic surveys of TB patients for the period 2003–2017 were reviewed. Isoniazid data were available from 156 countries or territories for 211,753 patients. Among these, the global prevalence of Hr-TB was 7.4% (95% CI 6.5%–8.4%) among new TB patients and 11.4% (95% CI 9.4%–13.4%) among previously treated TB patients. Additional data on pyrazinamide and levofloxacin resistance were available from 6 countries (Azerbaijan, Bangladesh, Belarus, Pakistan, the Philippines, and South Africa). There were no cases of resistance to both pyrazinamide and levofloxacin among Hr-TB patients, except for the Philippines (1.8%, 95% CI 0.2–6.4) and Belarus (5.3%, 95% CI 0.1–26.0). Sequencing data for all genomic regions involved in isoniazid resistance were available for 4,563 patients. Among the 1,174 isolates that were resistant by either phenotypic testing or sequencing, 78.6% (95% CI 76.1%–80.9%) had resistance-conferring mutations in the *katG* gene and 14.6% (95% CI 12.7%–16.8%) in both *katG* and the *inhA* promoter region. For 6.8% (95% CI 5.4%–8.4%) of patients, mutations occurred in the *inhA* promoter alone, for whom an increased dose of isoniazid may be considered. The main limitations of this study are that most analyses were performed at the national rather than individual patient level and that the quality of laboratory testing may vary between countries.

**Conclusions:**

In this study, the prevalence of Hr-TB among TB patients was higher than the prevalence of rifampicin resistance globally. Many patients with Hr-TB would be missed by current diagnostic algorithms driven by rifampicin testing, highlighting the need for new rapid molecular technologies to ensure access to appropriate treatment and care. The low prevalence of resistance to pyrazinamide and fluoroquinolones among patients with Hr-TB provides further justification for the recommended modified treatment regimen.

## Abstract 2

Anna Dean and colleagues study patterns of isoniazid resistance in tuberculosis patients across 156 countries or territories.

## Author summary

**Why was this study done?:**

- Tuberculosis (TB) is the leading cause of death from an infectious disease, with 1.2 million deaths occurring in 2018.
- People infected with a TB strain that is resistant to standard medicines used for treatment, including rifampicin or isoniazid, have a lower chance of being cured and a higher risk of death.
- In 2018, the World Health Organization (WHO) recommended a modified treatment regimen for patients with TB that is resistant to isoniazid but susceptible to rifampicin (Hr-TB).

**What did the researchers do and find?:**

- Data reported from 156 countries or territories to WHO between 2002 and 2018 were used to determine that a relatively high proportion of TB patients had Hr-TB: 7.4% (95% CI 6.5%–8.4%) of new patients and 11.4% (95% CI 9.4%–13.4%) of previously treated patients.
- In 6 countries with testing results for other medicines that are recommended in the modified treatment regimen (pyrazinamide and levofloxacin), resistance among Hr-TB patients was uncommon. Combined resistance to both drugs was only detected in the Philippines and Belarus but not in Azerbaijan, Bangladesh, Pakistan, or South Africa.
- Data from whole genome sequencing were also used to investigate markers of isoniazid resistance in 6 countries (Azerbaijan, Belarus, Bangladesh, Pakistan, South Africa and Ukraine).

**What do these findings mean?:**

- The modified treatment regimen recommended by WHO in 2018 for Hr-TB patients is likely to be effective in many settings.
- However, many patients with Hr-TB are missed because of the lack of laboratory capacity in many countries to screen for isoniazid resistance and the absence of appropriate diagnostic tools.

## Introduction

In 2018, an estimated 10 million people developed tuberculosis (TB) globally, and TB was the leading cause of death from a single infectious agent with an estimated 1.2 million deaths [1]. The surveillance of drug resistance among TB patients is central to combatting the global TB epidemic and preventing the spread of antimicrobial resistance. Surveillance data can be used to guide the planning of TB diagnostic and treatment services, design appropriate treatment regimens, and monitor the effectiveness of interventions.

The Global Project on Anti-TB Drug Resistance Surveillance, hosted by the World Health Organization (WHO), is the oldest and largest antimicrobial resistance surveillance project in the world [2]. Since its launch in 1994, data on drug resistance among TB patients have been systematically collected and analysed from 164 countries worldwide, collectively accounting for more than 99% of the world’s population and TB patients. This includes 105 countries that have continuous surveillance systems based on routine diagnostic drug susceptibility testing of most TB patients and 59 countries that rely on periodic epidemiological surveys of nationally representative samples of patients.

Rifampicin-resistant TB requires treatment with a second-line treatment regimen [3]. Using data from the Global Project, 3.4% (95% CI 2.5%–4.4%) of new TB cases and 18% (95% CI 7.6%–31%) of previously treated TB cases were estimated to have rifampicin-resistant TB in 2018. This includes multidrug-resistant (MDR)-TB, which is defined as resistance to both rifampicin and isoniazid. In 2018, 484,000 incident cases of rifampicin-resistant TB were estimated to have occurred worldwide [1].

Together with rifampicin, isoniazid is one of the most powerful drugs for the treatment of TB. These 2 drugs represent the cornerstone of the first-line treatment regimen recommended by WHO and used worldwide [4]. It is also a component of both shorter and longer second-line treatment regimens for rifampicin-resistant TB [3]. Isoniazid is critical not only to the treatment of active TB, but it is also the most commonly used medicine for preventive therapy [5,6].

Concerningly, a recent systematic review of published literature revealed that treatment of isoniazid-resistant, rifampicin-susceptible TB (Hr-TB) with the standard first-line regimen for new TB patients resulted in 11% (95% CI 6%–17%) treatment failure, compared with 2% (95% CI 1%–3%) among drug-susceptible TB patients [7]. To increase the likelihood of long-term cure of TB patients, WHO released updated guidance for the treatment of patients with Hr-TB in 2018 [8]. The main regimen is a 6-month course of rifampicin, pyrazinamide, ethambutol, and levofloxacin. The addition of a higher dose of isoniazid may be considered if only low-level isoniazid resistance is present, as indicated by specific *inhA* promoter mutations in the absence of *katG* mutations.

In this article, we describe the global prevalence of Hr-TB, with and without resistance to other anti-TB medicines critical for its treatment, and molecular markers of isoniazid resistance. We also discuss the implications of our findings for TB diagnosis, treatment, and care.

## Methods

### Study population

This study is reported as per the Strengthening the Reporting of Observational Studies in Epidemiology (STROBE) guideline (S1 STROBE Checklist). While no prospective protocol was developed, the analysis was conducted in line with standard methodology used to generate the prevalence estimates that are presented each year in WHO’s Global TB Report [1]. The analysis was conducted following release of the 2018 edition of the Global TB Report in October 2018 [9].

Aggregated data—from either routine continuous surveillance or national representative periodic surveys—on the proportions of new and previously treated cases with resistance to rifampicin and isoniazid are reported annually by countries to WHO. For surveillance data to be considered nationally representative, at least 80% of notified cases of pulmonary laboratory-confirmed TB must have a documented test result for drug susceptibility for at least rifampicin [1]. Countries without such continuous surveillance systems rely on nationally representative periodic surveys to estimate the burden of drug-resistant TB, for which methods are described elsewhere [10]. The focus of these surveys is the measurement of resistance to rifampicin and isoniazid, although data on resistance to other drugs may also be collected.

For 6 countries in which surveys were conducted between 2010 and 2014, representative data on resistance to pyrazinamide and fluoroquinolones were also available for both new and previously treated Hr-TB patients. Details of these surveys are provided elsewhere [11]. The 6 countries represented a range of programmatic and epidemiological settings and included Azerbaijan [12], Bangladesh [13], Belarus (city of Minsk) [14], Pakistan [15], the Philippines [16], and South Africa (Gauteng and Kwazulu Natal provinces) [17]. Data on molecular markers of isoniazid resistance were also available from these countries, as well as for Ukraine [18]. For each survey, ethical approval and written informed consent from patients was obtained (S1 Text).

### Laboratory analyses

#### Isoniazid and rifampicin

Testing for isoniazid and rifampicin in surveys and continuous surveillance uses either molecular or phenotypic methods. For the 7 countries mentioned, culture isolates from enrolled patients were tested using phenotypic methods according to the most recent WHO recommendations at the time [19], by either the Löwenstein-Jensen proportion method (Azerbaijan, Bangladesh, Pakistan, the Philippines, and Ukraine) or MGIT 960 (Becton Dickinson, Sparks, MD, US; Belarus and South Africa) at the following concentrations: 0.2 mg/mL isoniazid and 40.0 mg/L rifampicin on Löwenstein-Jensen medium and 0.1 mg/mL isoniazid and 1.0 mg/L rifampicin on MGIT 960.

#### Fluoroquinolones

Testing for fluoroquinolones (moxifloxacin and levofloxacin) was done using phenotypic methods according to the most recent WHO recommendations at the time [19]. For 5 countries (Azerbaijan, Bangladesh, Belarus, Pakistan, and South Africa), all isolates were tested for moxifloxacin at 0.5 mg/L using MGIT 960. Isolates that were resistant to moxifloxacin were tested for resistance to levofloxacin at 1.5 mg/L using MGIT 960. Isolates that were susceptible to moxifloxacin at 0.5 mg/mL were assumed to be susceptible to levofloxacin at 1.5 mg/L. For the Philippines, drug susceptibility testing was performed on a randomly selected subset of isolates for levofloxacin at 1.5 mg/L using MGIT 960. Drug susceptibility testing for levofloxacin was not performed for Ukraine.

#### Sequencing

Whole genome sequencing was performed for 6 countries (Azerbaijan, Bangladesh, Belarus, South Africa, Ukraine, and a subset of isolates from Pakistan). Illumina technology (Illumina, San Diego, CA, US) was used for all countries except for Belarus, where Ion Torrent technology (Thermo Fisher Scientific, MA, US) was used. The following extended promoter and/or coding regions were considered for isoniazid: *ahpC* (Rv2428), *ahpC* upstream, *fabG*, *inhA*, *inhA* promoter (*Rv1483*, *fabG1* upstream region), *katG*, *furA* (*Rv1909c*), and *furA* upstream. Targeted gene sequencing was performed for 2 countries (the Philippines and most of the Pakistan isolates) using Sanger technology (Thermo Fisher Scientific) for the relevant regions of *inhA* (locus *Rv1484*), *inhA* promoter, and *katG* (*Rv1908c*) genes. For pyrazinamide, the *pncA* (*Rv2043c*) gene was considered. For fluoroquinolones, the *gyrA* and *gyrB* genes were considered.

An established framework was used for the classification of detected mutations according to the confidence with which they are associated with drug resistance [20]. Mutations classified as being associated with resistance to rifampicin, isoniazid, or fluoroquinolones were considered to be conferring true resistance, even if phenotypic testing showed susceptibility. Laboratory methods have been published in detail elsewhere [11,21], and whole genome sequencing data are available on the Sequence Read Archive of the National Center for Biotechnology Information (https://www.ncbi.nlm.nih.gov/sra) as recalibrated BAM files (accession number SRP128089).

### Statistical analysis

Using the most recent year of data available from the 17-year period from 2002 to 2018, the proportions of new and previously treated TB cases with resistance to anti-TB medicines were estimated for each country, for the 6 WHO regions, and globally for the following 3 resistance patterns: (i) isoniazid resistance without rifampicin resistance (Hr-TB), (ii) any isoniazid resistance, and (iii) concurrent isoniazid and rifampicin resistance (MDR-TB). Weights corresponding to the number of notified new and previously treated pulmonary TB cases in each country in 2017 were applied [1]. The distributional shape of the data was captured in violin plots. Countries without data for both rifampicin and isoniazid were excluded from global and regional estimations.

The prevalence of resistance to levofloxacin (from phenotypic testing, adjusted by sequencing) and pyrazinamide (from sequencing) was calculated for new and previously treated Hr-TB cases in Azerbaijan, Bangladesh, Belarus, Pakistan, the Philippines, and South Africa. For isolates from countries with whole genome sequencing data (i.e., Azerbaijan, Bangladesh, Belarus, South Africa, Ukraine, and a subset of the Pakistan isolates), the frequency of mutations known to confer resistance to isoniazid was calculated among samples displaying either phenotypic and/or genotypic isoniazid resistance.

## Results

### Epidemiological data

**Fig 1:**
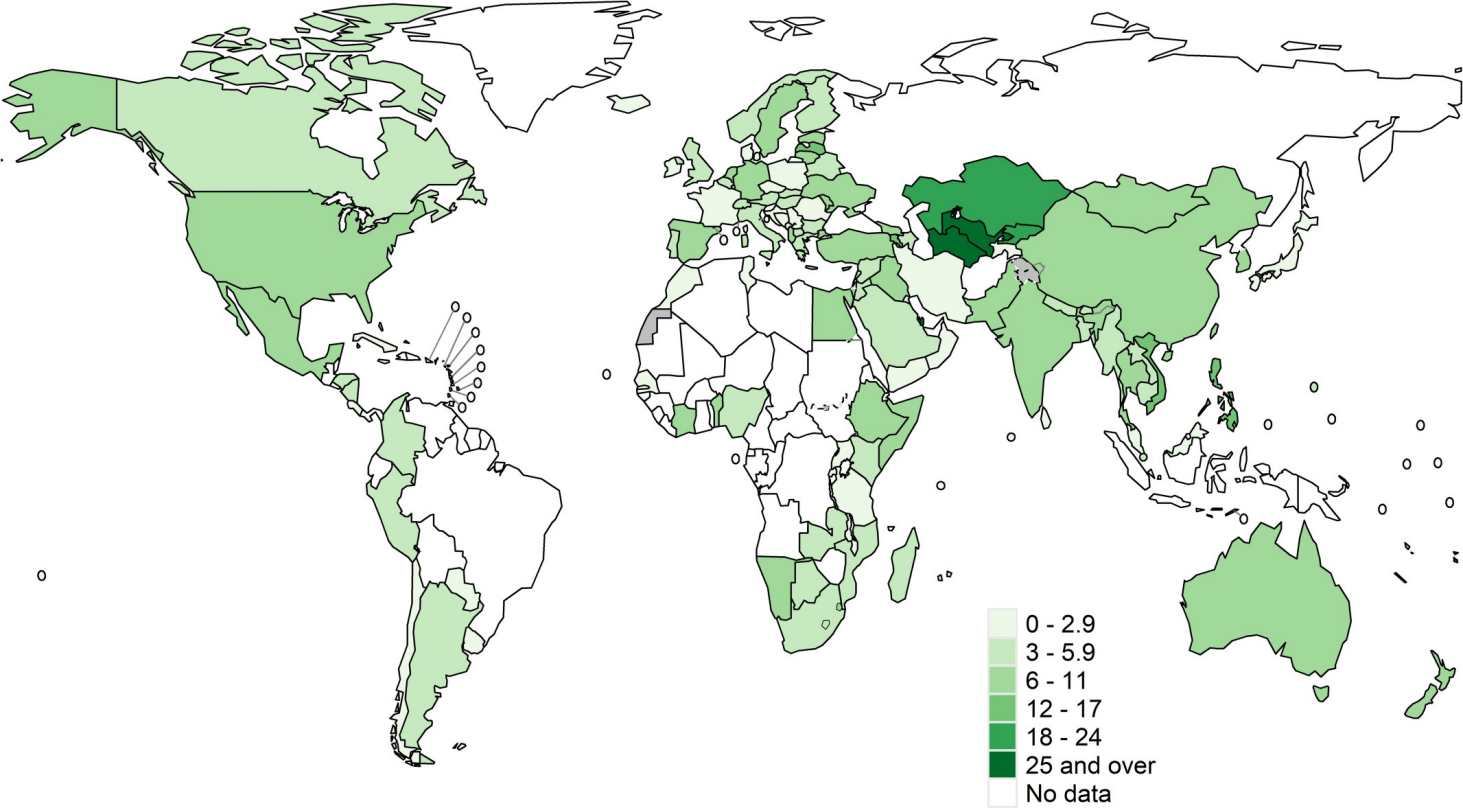
Prevalence of Hr-TB among new TB cases. These maps were created using the R package, “whomap.” Hr-TB, isoniazid-resistant, rifampicin-susceptible TB; TB, tuberculosis.

**Fig 2:**
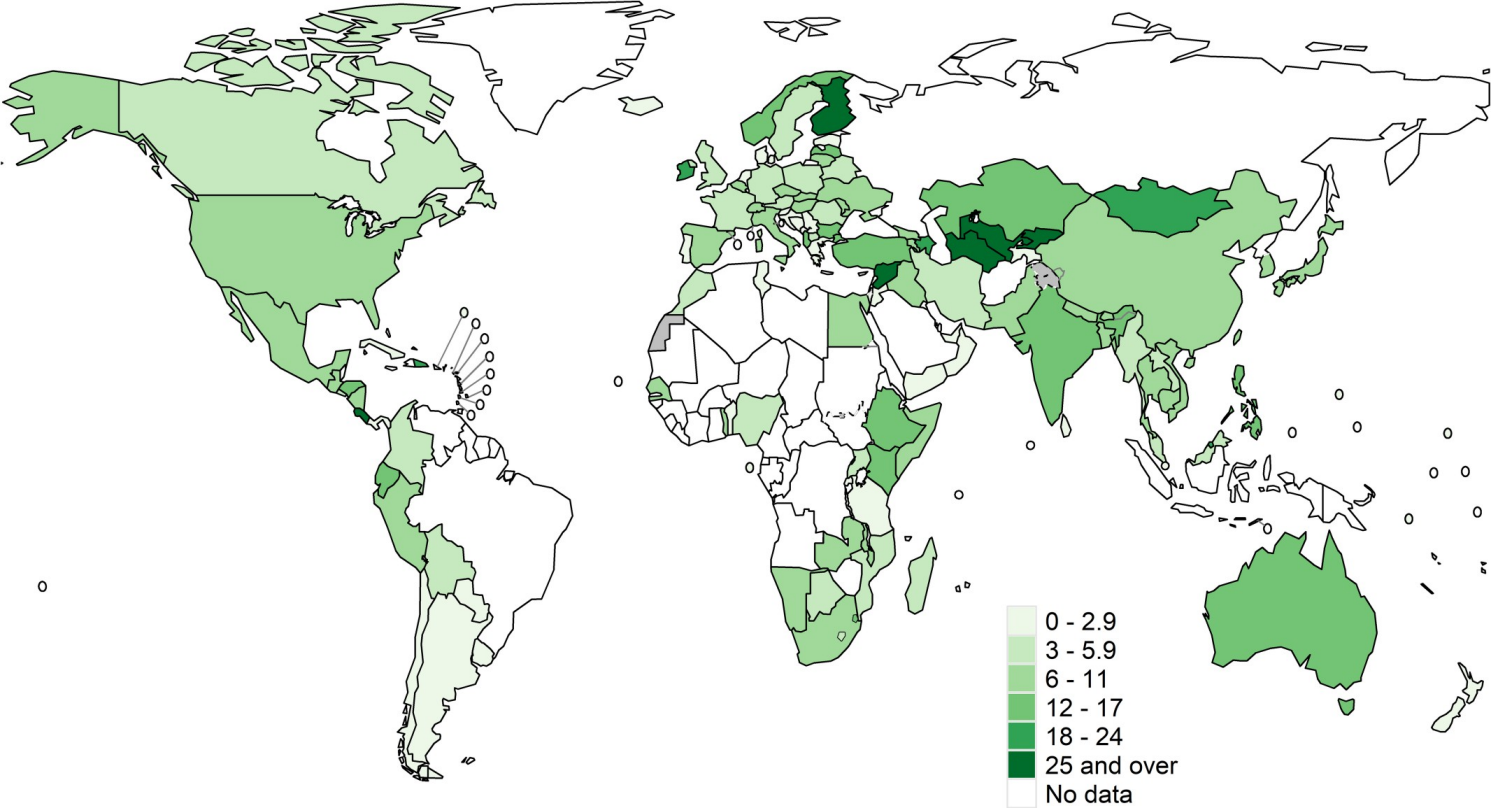
Prevalence of Hr-TB among previously treated TB cases. These maps were created using the R package, “whomap.” Hr-TB, isoniazid-resistant, rifampicin-susceptible TB; TB, tuberculosis.

**Fig 3:**
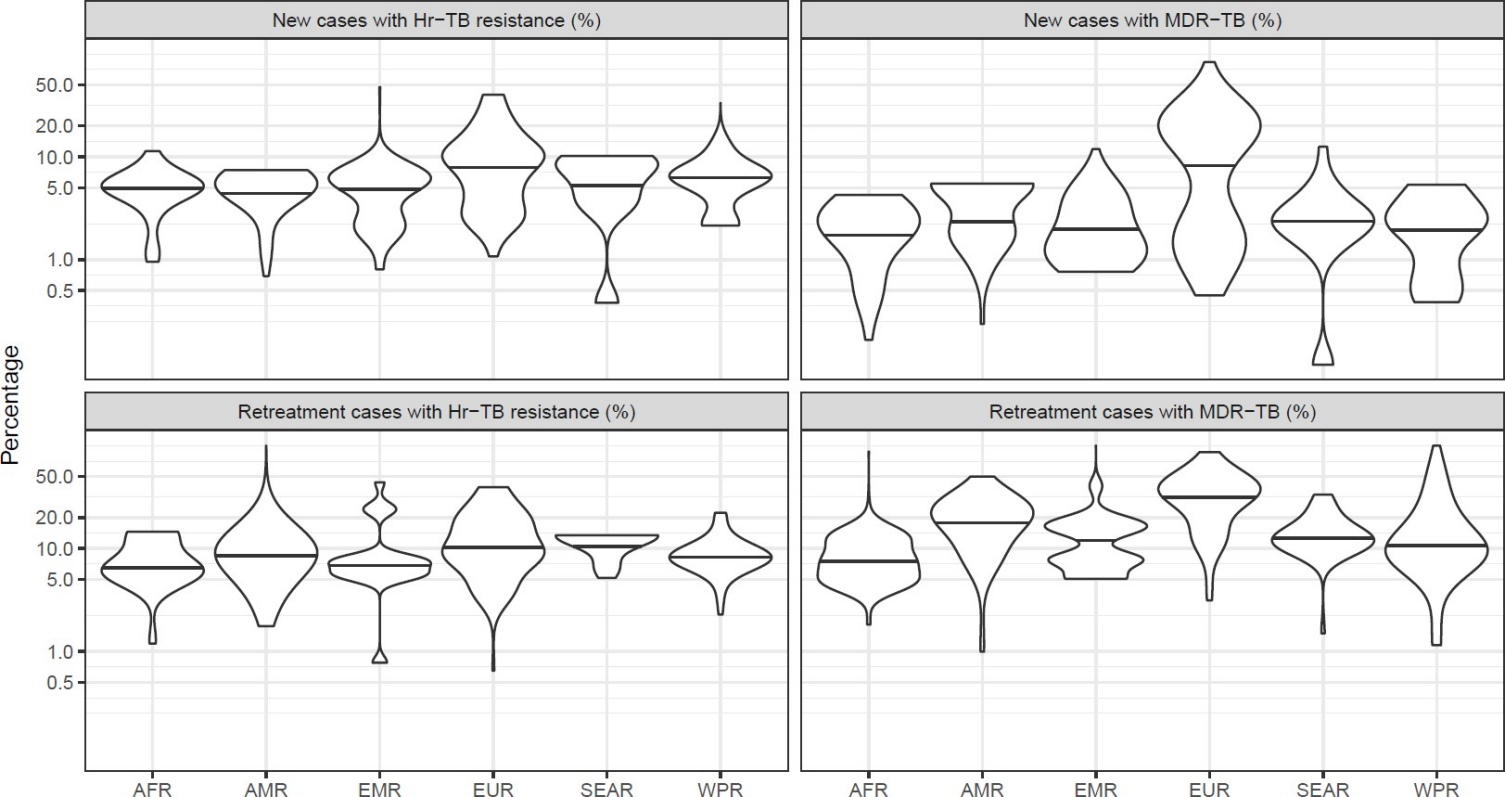
Prevalence (%) of Hr-TB and MDR-TB among new and previously treated TB cases by WHO region. The median national prevalence within each region is indicated by a horizontal line. AFR, African Region; AMR, American Region; EMR, Eastern Mediterranean Region; EURO, European Region; Hr-TB, isoniazid-resistant, rifampicin-susceptible TB; MDR, multidrug-resistant TB; SEAR, Southeast Asian Region; TB, tuberculosis; WPR, Western Pacific Region.

Isoniazid data were available from 156 countries or territories for 211,753 pulmonary TB patients from 2002 to 2018 (S1 Data). Global maps of the prevalence of resistance among new cases are shown in Fig 1 and for previously treated cases in Fig 2. Among the countries and territories for which data were available, the global prevalence of Hr-TB was 7.4% (95% CI 6.5%–8.4%) among new TB patients, 11.4% (95% CI 9.4%–13.4%) among previously treated TB patients (Table 1), and 8.4% (95% CI 7.5%–9.2%) among all TB patients. The distributions of the national prevalence of Hr-TB and MDR-TB displayed significant variance within each WHO region (Fig 3). The difference in the median prevalence of Hr-TB for new compared to previously treated cases was smaller for Hr-TB than for MDR-TB across all regions (S2 Data). Globally, the prevalence of any resistance to isoniazid—regardless of rifampicin resistance status—was 10.7% (95% CI 9.6%–11.9%) among new TB patients and 27.2% (95% CI 24.6%–29.9%) among previously treated TB patients (Table 1).

**Table 1:** Prevalence (%) of isoniazid resistance among new and previously treated TB cases by WHO region.

	Isoniazid resistance without rifampicin resistance (Hr-TB)		Any isoniazid resistance	
WHO region	New	Previously treated	New	Previously treated
Africa	4.5 (3.5–5.5)	6.3 (3.1–10.4)	6.4 (5.2–7.6)	13.5 (8.8–19.0)
Americas	5.0 (4.1–6.1)	7.6 (5.3–10.3)	8.0 (6.9–9.2)	22.8 (28.9–36.8)
Eastern Mediterranean	7.2 (5.8–8.7)	6.3 (3.3–10.2)	10.7 (9.1–12.5)	23.5 (17.8–29.7)
European	4.9 (3.7–6.2)	15.3 (13.9–16.8)	25.4 (24.0–26.7)	47.4 (45.4–49.3)
Southeast Asian	7.6 (6.6–8.6)	12.6 (11.1–14.3)	10.3 (9.2–11.4)	25.7 (23.7–27.8)
Western Pacific	8.8 (8.0–9.6)	11.5 (7.4–16.4)	12.9 (12.0–13.9)	33.9 (27.9–40.2)
Global	7.4 (6.5–8.4)	11.4 (9.4–13.4)	10.7 (9.6–11.9)	27.2 (24.6–29.9)

**Abbreviations:** Hr-TB, isoniazid-resistant, rifampicin-susceptible TB; TB, tuberculosis; WHO, World Health Organisation

Among Hr-TB cases, the prevalence of resistance to either levofloxacin or pyrazinamide was low (Table 2). An exception was Pakistan, where 12.6% of new Hr-TB patients had resistance to at least one of these drugs. There were no cases of resistance to both drugs among new Hr-TB patients in Azerbaijan, Bangladesh, Pakistan, or South Africa. In the Philippines, 1.8% (95% CI 0.2%–6.4%) of new Hr-TB patients had resistance to both drugs. In Belarus, 5.6% (95% CI 0.1%–27.3%) of new Hr-TB patients had resistance to both drugs, but the sample size was small (only 19 new Hr-TB cases).

**Table 2:** Prevalence of resistance to levofloxacin and pyrazinamide among new and previously treated isoniazid-resistant, rifampicin-susceptible TB (Hr-TB) cases.

	Azerbaijan	Bangladesh	Belarus (city of Minsk)	Pakistan	Philippines	South Africa (Gauteng)	South Africa (KwaZulu Natal)
**Levofloxacin 1.5 mg/L**							
New	0/60, 0%	0/37, 0%	1/19, 5.3%	13/112, 11.6%	0/165, 0%	0/39, 0%	0/19, 0%
	(0%–6.0%)	(0%–9.5%)	(0.1%–26.0%)	(6.3%–19.0%)	(0%–2.2%)	(0%–9.0%)	(0%–17.6%)
Previously treated	2/44, 4.5%	0/10, 0%	1/2, 50%	4/14, 28.6%	0/29, 0%	0/10, 0%	0/14, 0%
	(0.6%–15.5%)	(0%–30.8%)	(1.3%–98.7%)	(8.4%–58.1%)	(0%–11.9%)	(0%–30.8%)	(0%–23.2%)
**Pyrazinamide (pncA sequencing)**							
New	3/62, 4.8%	0/38, 0%	1/20, 5.0%	1/111, 0.9%	2/111, 1.8%	0/37, 0%	0/19, 0%
	(1.0%–13.5%)	(0%–9.3%)	(0.1%–24.9%)	(0%–4.9%)	(0.2%–6.4%)	(0%–9.5%)	(0%–17.6%)
Previously treated	3/43, 7.0%	0/10, 0%	1/3, 33.3%	0/13, 0%	1/9, 11.1%	0/10, 0%	0/14, 0%
	(1.5%–19.1%)	(0%–30.8%)	(0.8%–90.6%)	(0%–24.7%)	(0.3%–48.2%)	(0%–30.8%)	(0%–23.2%)
**Any resistance to levofloxacin 1.5 mg/L or pyrazinamide (pncA sequencing)**							
New	3/60, 5.0%	0/37, 0%	1/19, 5.3%	14/111, 12.6%	2/110, 1.8%	0/35, 0%	0/19, 0%
	(1.0%–13.9%)	(0%–9.5%)	(0.1%–26.0%)	(7.0%–20.3%)	(0.2%–6.4%)	(0%–10.0%)	(0%–17.6%)
Previously treated	3/43, 7.0%	0/10, 0%	1/2, 50%	4/13, 30.8%	1/9, 11.1%	0/10, 0%	0/14, 0%
	(1.5%–19.1%)	(0%–30.8%)	(1.3%–98.7%)	(9.1%–61.4%)	(0.3%–48.2%)	(0%–30.8%)	(0%–23.2%)
**Combined resistance to levofloxacin 1.5 mg/L and pyrazinamide (pncA sequencing)**							
New	0/60, 0%	0/37, 0%	1/19, 5.3%	0/111, 0%	0/110, 0%	0/35, 0%	0/19, 0%
	(0%–6.0%)	(0%–9.5%)	(0.1%–26.0%)	(0%–3.3%)	(0%–3.3%)	(0%–10.0%)	(0%–17.6%)
Previously treated	1/43, 2.3%	0/10, 0%	1/2, 50.0%	0/13, 0%	0/9, 0%	0/10, 0%	0/14, 0%
	(0.1%–12.3%)	(0%–30.8%)	(1.3%–98.7%)	(0%–24.7%)	(0%–33.6%)	(0%–30.8%)	(0%–23.2%)

Data shown are number of patients resistant divided by number tested, proportion resistant (%) (95% confidence interval).

**Abbreviations:** Hr-TB, isoniazid-resistant, rifampicin-susceptible TB; TB, tuberculosis

### Molecular analyses

Whole genome sequencing data were available from culture isolates from 4,795 patients from 6 countries (Azerbaijan, Bangladesh, Belarus, Pakistan, Ukraine and South Africa) [21]. Of these, 4,564 had complete coverage of all genomic regions involved in mechanisms of resistance to isoniazid, and 4,559 also had phenotypic testing results available. Of these 4,559 patients, 1,174 were isoniazid resistant by either phenotypic testing or sequencing (S3 Data). According to the established framework for mutation classification [20], high confidence resistance-conferring mutations were identified in the *katG* gene in 923 (78.6%, 95% CI 76.1%–80.9%) of isoniazid-resistant isolates, all occurring at codon 315 (Ser315Asn or Ser315Thr). For 751 (64%, 95% CI 61.1%–66.7%) of isoniazid-resistant isolates, these mutations occurred in the *katG* gene alone, while 172 (14.6%, 95% CI 12.7%–16.8%) of isoniazid-resistant isolates had concurrent resistance-conferring mutations in the *inhA* promoter region. Resistance-conferring mutations in the *inhA* promoter region alone were detected in an additional 80 (6.8%, 95% CI 5.4%–8.4%) isoniazid-resistant isolates, all of which were c-15t. Among the 1,174 isolates that were isoniazid resistant by either phenotypic testing or sequencing, mutations that were not yet graded as being associated with resistance according to the established framework—but have nonetheless been previously reported among phenotypically resistant strains—were identified in 109 patients (9.3%, 95% CI 7.7%–11.1%). Such mutations were found in the *inhA* promoter and coding, *katG* coding, *fabG* coding, or *ahpC* promoter regions. For 52 isolates (4.4%; 95% CI 3.3%–5.8%), these mutations occurred in in the absence of any other graded mutations.

## Discussion

In this study, we used aggregated data reported to WHO from 156 countries or territories in 2002–2018 to estimate the prevalence of Hr-TB among new and previously treated TB patients. For 6 countries (Azerbaijan, Bangladesh, Belarus, Pakistan, the Philippines, and South Africa) with results from further phenotypic drug susceptibility testing as well as data from whole genome or targeted sequencing, the prevalence of resistance to pyrazinamide and levofloxacin was estimated among Hr-TB patients. For 6 countries with whole genome sequencing data (Azerbaijan, Bangladesh, Belarus, Pakistan, South Africa and Ukraine), molecular markers of isoniazid resistance were investigated.

Among countries for which data were available in our study, the global prevalence of Hr-TB among new cases was 7.4% (95% CI 6.5%–8.4%) and among previously treated cases was 11.4% (95% CI 9.4%–13.4%). The wide variability in the prevalence of Hr-TB and MDR-TB between countries within the same region highlights the important, yet poorly understood, role of setting-specific factors on the emergence and spread of rifampicin and isoniazid resistance. Potential associations with HIV could not be explored, as these data were not available. The prevalence of Hr-TB in our study is higher than the global estimate of the prevalence of rifampicin resistance, which includes MDR-TB [1]. Given the lower and narrower confidence bounds around the prevalence estimate for Hr-TB, a history of previous treatment may be less important when screening for potential Hr-TB as opposed to rifampicin-resistant TB.

To our knowledge, this study represents the first multicountry assessment of resistance to other TB medicines among over 1,000 Hr-TB patients and provides further justification for the recommended treatment regimen. There were no cases of combined resistance to pyrazinamide and levofloxacin among new Hr-TB patients in Azerbaijan, Bangladesh, Pakistan, the Philippines, or South Africa, although the prevalence of resistance to each drug varied between settings. In Pakistan, the prevalence of levofloxacin resistance (10.1% among new cases, 95% CI 6.7%–13.4%) was alarmingly high compared to the prevalence of rifampicin resistance (4.2% among new cases, 95% CI 3.2%–5.3%) [11]. This likely relates to the indiscriminate use of fluoroquinolones for the treatment of TB as well as pneumonia and uncomplicated respiratory tract infections. Patients with resistance to levofloxacin may require an individually tailored regimen.

Diagnosing people with Hr-TB remains a challenge. A large number of TB patients eligible for the modified treatment regimen for Hr-TB recommended by WHO in 2018 [8] are missed by current algorithms for drug susceptibility testing. In many countries, diagnostic algorithms are driven by the combined testing for TB and rifampicin resistance offered by the GeneXpert (Cepheid, CA, US) platform, which allows rapid molecular testing at decentralized levels. Rifampicin testing is used as an initial screening tool to identify patients requiring further drug susceptibility testing and those for whom a rifampicin-resistant TB treatment regimen is indicated [3]. While the use of this technology is justified, the absence of a similar tool to detect Hr-TB means that a proportion of TB patients may continue to receive suboptimal first-line regimens, experience poorer treatment outcomes, and develop further resistance to other drugs. Line probe assays, such as the WHO-endorsed Hain MDRTB*plus* (Hain Lifesciences, Nehren, Germany) and the Nipro NTM+MDRTBII (Nipro, Osaka, Japan), reliably detect mutations in the *katG* gene and *inhA* promoter region, which account for more than 85% of isoniazid-resistant cases in our dataset. Therefore, while tests with good accuracy do already exist, they cannot be easily integrated into routine diagnostic algorithms in many countries. The development of a simple, rapid, accurate, and affordable test to detect isoniazid resistance at the peripheral level is urgently required. Encouragingly, the TB diagnostic landscape continues to expand, and several promising technologies are currently in the development or evaluation phase [22].

To better understand the role of mutations in conferring isoniazid resistance, more data are needed from next generation sequencing and phenotypic testing. Double mutants (the presence of resistance-conferring mutations in codon 315 of the *katG* gene and in the *inhA* promoter region) are known to display high-level resistance to isoniazid [23]. Among isolates with whole genome sequencing data for extended isoniazid targets, only 14.6% (95% CI 12.7%–16.8%) of 1,174 isolates with either phenotypic and/or genotypic resistance were double mutants. However, an additional 64% of the 1,174 isolates had mutations in codon 315 of the *katG* gene (alone or in the presence of an ungraded mutation in the *inhA* promoter), which confers at least a moderate level of resistance. For patients with moderate or high levels of resistance (78.6%, 95% CI 76.1%–80.9%), increasing the dose of isoniazid is likely not an option. However, when specific *inhA* promoter mutations are detected in the absence of codon 315 *katG* mutations, in vitro evidence suggests that increasing the dose of isoniazid may be effective [8]. This may be appropriate for 6.8% (95% CI 5.4%–8.4%) of isoniazid-resistant patients in our dataset. Mutations that have not been graded in the adopted system may indeed confer resistance, but current evidence is insufficient to demonstrate a statistically significant association. Ungraded mutations were detected in 9.3% (95% CI 7.7%–11.1%) of isoniazid-resistant isolates. Some of these isolates also had concurrent graded mutations, or other ungraded mutations that are still detected by the line probe assay (e.g., *inhA* promoter −8 region). Some patients risk being incorrectly classified as having isoniazid-susceptible TB, or as having only low-level isoniazid resistance if other mutations are present in the *inhA* promoter region [24].

Our analysis has 3 main limitations. First, it was based primarily on aggregated, cross-sectional data, rather than individual case-based data, from 2 different sources. Countries without available data were excluded from the analysis. Periodic surveys may have more robust quality management systems than routine continuous surveillance, and therefore these data may have less risk of bias. To improve data quality and allow an analysis of trends over time, countries should move towards establishing electronic, case-based recording and reporting surveillance systems for TB coupled with quality-assured laboratory testing. Second, although case-based data were available for 7 high–TB-burden countries from different epidemiological settings, they were not linked to information about treatment regimens or outcomes, which could have allowed a better understanding of their clinical relevance. Third, the quality of phenotypic and genotypic testing for rifampicin and isoniazid may vary between countries, resulting in misclassification of cases, which could bias estimates of the prevalence of resistance. The critical concentrations used for phenotypic testing of levofloxacin was higher than the concentration now recommended by WHO in new guidance issued in 2018 [19]. However, possible underestimation of levels of resistance was mitigated by taking into account the sequencing results when classifying isolates as resistant or susceptible. The framework for classification of detected mutations continues to be regularly updated using the latest available data.

## Conclusion

Isoniazid-resistant TB poses a challenge to global efforts for ending the TB epidemic by 2030. This study shows that many patients with Hr-TB would be missed by current diagnostic algorithms driven by rifampicin testing, highlighting the need for new rapid molecular technologies to ensure access to appropriate treatment and care. The low prevalence of resistance to pyrazinamide and fluoroquinolones among patients with Hr-TB provides further justification for the recommended modified treatment regimen. The analysis of drug resistance patterns should ideally be linked to patient treatment outcome data, to better understand the clinical relevance of laboratory testing results and contribute to global policy for clinical decision-making.

## Supporting information

S1 STROBE Checklist Completed STROBE checklist. STROBE, Strengthening the Reporting of Observational Studies in Epidemiology.
(DOCX)

S1 Text National ethics approvals received. (DOCX)

S1 Data Prevalence of Hr-TB and MDR-TB by country for new and previously treated patients. Hr-TB, isoniazid-resistant, rifampicin-susceptible tuberculosis; MDR-TB, multidrug-resistant tuberculosis.
(XLSX)

S2 Data Median prevalence of Hr-TB and MDR-TB by region for new and previously treated patients. Hr-TB, isoniazid-resistant, rifampicin-susceptible tuberculosis; MDR-TB, multidrug-resistant tuberculosis.
(XLSX)

S3 Data Analysis of isoniazid targets from whole genome sequencing. (XLSX)

## Abbreviations

HR-TB: isoniazid-resistant, rifampicin-susceptible TB
MDR-TB: multidrug-resistant TB
STROBE: Strengthening the Reporting of Observational Studies in Epidemiology
TB: tuberculosis
WHO: World Health Organization
