# Supplementary material for: Prevalence and genetic profiles of isoniazid resistance in tuberculosis patients: A multicountry analysis of cross-sectional data
Source: PLoS Med. 2020 Jan 21;17(1):e1003008. doi: 10.1371/journal.pmed.1003008 (PMC6974034; doi:10.1371/journal.pmed.1003008)
Supplement: S1 Text — (DOCX) [file pmed.1003008.s002.docx]

**Ethics approvals received**

Azerbaijan: Ministry of Health of the Republic of Azerbaijan

Bangladesh: Bangladesh Medical Research Council

Belarus: Republican Scientific and Practical Centre for Pulmonology and Tuberculosis

Pakistan: The Pakistan Medical Research Council

Philippines: Institutional Review Board of the Research Institute for Tropical Medicine

South Africa: University of Witwatersrand Research Ethics Committee
